# Healthy Houses

**Published:** 1857-05

**Authors:** 


### HEALTHY HOUSES.

RECENT occurrences in Washington City, prove the truth of an article we published two years ago, on "*Health and house hunting.*" It is estimated that not less than a thousand persons had their health seriously impaired while at the National Hotel, in consequence of the drainings of the immense establishment having been prevented from passing off, allowing no escape for their destructive effluvia, except upwards through the building, first saturating the meats which were eaten, and then the atmosphere which the guests were compelled to breathe every moment they were on the premises; thus, at least, reported.

There can be no doubt that millions of people die every year from similar causes, but being less concentrated, the work is done in too gradual a manner to excite suspicion. In Boston, a number of years ago, very special pains were taken to keep the city in an unexceptionable cleanly condition, to prevent the advent of cholera. Every privy, every back yard, every gutter, was scrupulously examined, and the occupant of each house was enjoined to keep the kitchen and pantry scrupulously free from dirt and dampness. Yet, after all this precaution, the cholera did appear in one street with great malignity, and a severe disappointment and discouragement was the result as to the efficacy of such sanitary measures. All was explained, however, when the visiting committee entered the cellar of an indicated house, and there, in its darkest corner, was the festering mass of corruption—the house offal of a whole winter. With its removal, the epidemic ceased.

If there is an architect in the city of New York or within a ten mile radius of the City Hall, who has three grains of common sense, let him be exhibited at the museum at a dime a head. It will pay largely.

What is the chief end of a modern Architect?

To make money.

What is his chief aim?

His chief aim is two-fold: To bring down the estimate to the lowest possible dollar, until the contract is closed, and then to pile up the "extras" by modifications and improvements to the utmost limit of endurance.

We do not believe that there is a single public building or private store or dwelling in New York, erected within ten years, that is not, in important features, a disgrace to the name of scientific architecture.

If there is in this city any public building erected within ten years, which does not leak, we would like to see it. Such would be an exception rather than the rule.

Does any observing man believe that there is in all New York and Philadelphia, a single safe house constructed to be warmed by furnace heat?

How many churches are there in the five largest cities in the Union which could be emptied of an ordinary congregation within three minutes, in case of sudden alarm of any kind? We enter ours—a type of all—through four successive doors, two of which open inwards. The large outer doors open inwards. Consequently, on a sudden alarm, there would be a jam, and no earthly power could open one of those doors; and in case of fire, multitudes would be burned to death standing in masses, as has been known to be the case at Richmond in our own country, and more recently, in one of the Russian cities. It is not safe to enter a single church or theatre or opera house in New York. The death-trap construction of the former *Tripler Hall*, built here for Jenny Lind, is fresh within the memory of all. Its destruction was the abatement of a nuisance. Had the incendiary fired it before the audience had dispersed, multitudes must have inevitably been burned to death or suffocated, notwithstanding it had been greatly altered from its original construction. But this, even, was owing to the demands of the daily press, and not to the philanthropy of the owner, or the good sense of the architect.

Look at the basement kitchens and dining-rooms of nine tenths of the dwellings of New York. Is it any wonder that our hospitals are filled with sick servant girls, and our cooks die off with *pneumonias*, *typhoid fever*, and *rheumatic disease?*

And that improvement in the Dartmoor prison line, the stately dwellings which, while they decorate our city, populate Greenwood, constructed "*three rooms deep*." Yes! that is the fashionable description of a house with all the modern improvements —"*three rooms deep*," the middle one being used as a bedroom, where, literally, a ray of sunshine never enters, and no air, but must come through another room from windows thirty feet away. Without question, our houses are made to die in, not to live in—not to be enjoyed. No room is fit for a bedroom which has not at least two windows facing the sun for several hours of every day.

Our churches should have all their doors opening in the direction of the street, or made to slide easily; besides, two large doors should be put in the part of the building opposite the main entrance, or side doors, which can be seen by the whole congregation, so that in a moment of alarm, there might be four directions of escape, for mind, a modern church window is too high; its sash is made of iron, and each light is often not large enough to get a good-sized head through. Hence, the only place for escape is through the doors.

As to dwellings "three rooms deep," so much the fashion now, they had better be demolished, or the centre rooms removed. And we recommend that if houses are not large enough, extensions should be made in the rear, in the form of what is technically called an "ell," which is the prevailing fashion in Philadelphia; and a most excellent and healthful arrangement it is, too, for by this means, every room is well exposed to the sun, causing light, purity, and dryness, the great essentials in any healthy dwelling. An impressive incident in illustration of this point, has recently come to light. A mercantile house doing a large business, observed in the course of years, that their bookkeepers died of consumption one after the other, however healthy they were on entering their employ. In looking around for a cause, it was discovered in the fact, that the room occupied had but one window, which looked upon a small back yard, enclosed on every side by buildings forty feet high, thus precluding every ray of sunshine. A change was instantly made. A light, airy apartment on the second or third floor was provided, and the trouble at once disappeared. A dog confined in a cellar will become consumptive in six weeks, according to the observation of medical men. No room without the glorious sunshine is fit for any living creature, man or beast. The glorious sunshine! The free and bounteous gift of a beneficent Creator—the source of all buoyant, healthful life. Yet in our truculence to fashion, in our greed of gold, in our infatuated indifference to the health of our wives, our children, and of ourselves, we remorselessly throw it all away, one of the loveliest gifts of a loving God, the beauteous sunshine!

As this May moving season is the time for changes, when many enter new purchased homes and all begin to "improve" more or less, we crave a mature consideration of the suggestions made, believing as we do, that it concerns the health of many families. And on all who change their habitations, we urge the bestowal of a large, a very large share of attention, first of all, to the cellar: remove every movable thing; open every door and hatchway; sweep it, yes, sweep it half a dozen times—floor, sides, ceiling; then give a plentiful coating of whitewash, made with unleached lime, and in all other respects, attend to the suggestions made a year ago, for the obvious reason, that whatever of filth is in the cellar, rises upwards and saturates the atmosphere of the whole building, not to kill you in a night not to poison your system in so short a space as a few days, as at the *National Hotel* in Washington City, but which in its more insidious workings, saps by slow degrees the health of those who are dearest to us, draining them of their vitality until none is left, and before we are aware, we find them a wreck, the mere shadow of what they were a few years before, in spite of their living in unexceptionable (outside) brown stone buildings, up town, in one of the best ventilated spots on the globe, with broad rolling rivers on either side, and an ocean at the foot, all owing to careless servants, their master setting the example, making the cellar the receptacle of all that is foul and filthy. There is more sound practical hygiene on this subject of healthy houses in the fourteenth chapter of *Leviticus*, from verse thirty-four, than in all the skulls of all the health commissioners and common councils of all the cities of Christendom. Pity it is that we don't read our Bible more- -that great book, which contains the leading principles of what is indisputably good, and useful, and true, in all that really pertains to human happiness; and pity is it, that the Sunday newspaper, and the trashy weekly, and the enticing story book, for childhood, youth, and hoary age, on subjects pertaining to the world, and party preaching, and infidel peripatetic lecturers, with their new-fangled crudities for human amelioration, and their inane theories for elevating the masses—pity is it, we say, that all these things so attract attention from day to day, that the Bible, the best book of all, and the wisest, true in all its theories, and in all its practices safe, has become a sealed book to the many, and any other volume on the centre or side table, is opened sooner than it. Oh! hie me to the "old paths" and to times of lang syne, when the Saturday afternoon Bible class was the thing talked of and prepared for during the week, its leader a William Wallace, and then a John McFarland, a pupil of the elder Mason; and these same youthful Bible learners now, the men of their generation, where are they? What are they doing? Why, they are scattered through this whole land, east and west, and in other lands, leading men everywhere, as secretaries, as professors, as presidents of colleges, as influential editors, clergymen of mark, and higher still, as missionaries to the distant heathen, and the privy counsellors of kings! Let us tell you, reader, a Bible man—a man whose principles are founded on Bible teachings—is a man everywhere, whether a shoe-black or an emperor—more, the only man who can be safely trusted, in all God's universe.

Let us then turn back to the BIBLE, one and all. Let us require that it, and it alone, should be read in our families on the Sabbath day, and not one line from tract, or sermon, or Sunday-school book, and we will all be the wiser, and better, and healthier, reasoning as it does of "*temperance*," enjoining "*cleanliness*," and teaching that the next virtue to "*godliness*," is "*contentment*," and in these three, contentment, cleanliness, and temperance, have we the essential elements of all practical hygiene.

